# Manufacturing of Lightweight Aggregates as an Auspicious Method of Sewage Sludge Utilization

**DOI:** 10.3390/ma13245635

**Published:** 2020-12-10

**Authors:** Jerzy Korol, Marcin Głodniok, Aleksander Hejna, Tomasz Pawlik, Błażej Chmielnicki, Jan Bondaruk

**Affiliations:** 1 Department of Material Engineering, Central Mining Institute, Pl. Gwarków 1, 40-166 Katowice, Poland; 2 Department of Water Protection, Central Mining Institute, Pl. Gwarków 1, 40-166 Katowice, Poland; mglodniok@gig.eu (M.G.); jbondaruk@gig.eu (J.B.); 3 Department of Polymer Technology, Gdańsk University of Technology, Narutowicza 11/12, 80-233 Gdańsk, Poland; aleksander.hejna@pg.edu.pl; 4 Faculty of Material Engineering and Metallurgy, Silesian University of Technology, Krasińskiego 8, 40-019 Katowice, Poland; Tomasz.Pawlik@polsl.pl; 5 Paint & Plastics Department in Gliwice, Institute for Engineering of Polymer Materials and Dyes, 50 A Chorzowska Street, 44-100 Gliwice, Poland; b.chmielnicki@impib.pl

**Keywords:** sewage sludge, lightweight aggregate, granulation, sintering, recycling

## Abstract

Sewage sludge is a high-volume and low-cost waste commonly generated worldwide, so its utilization is a vital issue. The application of this waste in the manufacturing of lightweight aggregates was investigated. The process was performed using intensive mixers with volumes of 5 and 30 L, as well as the industrial 500 L mixer. Then, granulates were sintered in a tube furnace. The influence of composition and mixer size on the particle size, microstructure, mechanical performance, and stability of lightweight aggregates in different environments was analyzed. The best results were obtained for a 500 L mixer, enhancing the industrial potential of the presented process. Increasing the share of sewage sludge in the composition of aggregates enhanced their porosity and reduced the specific weight, which caused a drop in compressive strength. Nevertheless, for all analyzed materials, the mechanical performance was superior compared to many commercial products. Therefore, sewage sludge can be efficiently applied as a raw material for the manufacturing of lightweight aggregates. The presented results confirm that a proper adjustment of composition allows easy the tailoring of aggregates’ performance and cost.

## 1. Introduction

Wastewater treatment plants are one of the sources of organic waste. The amount of sewage sludge produced in the European Union per year was 10 million tonnes in 2008, 11.5 million tonnes in 2015, and is expected to approach 13 million tonnes of dry matter (DM) by the end of 2020 [1]. Sewage sludge (SS) produced in Poland is expected to reach 746 Gg of dry weight per year by 2022 [2]. The primary method of SS disposal in Poland is through land-filling and agricultural use methods, and this practice will ultimately lead to land scarcity problems. Before SS disposal, it is usually fermented in fermentation tanks with the extraction of biogas and treated with chemicals such as lime, magnesium oxide, etc. [2]. The biological treatment of municipal wastewaters yields large amounts (up to 2% of the influent stream volume) of sewage sludges of different types (primary, secondary, mixed, and excess sludges), which are, in fact, waste organic–mineral by-products. They must undergo further multistage processing to eliminate ecological risks and microbiological hazards [3]. For these purposes, several physicochemical and biological processes can be applied, namely sludge thickening, dewatering, drying, pasteurization, irradiation, lime treatment, thermal stabilization by incineration, pyrolysis or gasification, conditioning, recovery of biogenic elements, aerobic composting, and finally, anaerobic digestion [4,5,6]. In particular, stabilization in anaerobic digesters followed by dewatering yields the waste sludge, code 19 08 05. Generally, the utilization of sewage sludge is a very popular topic among researchers all over the world. Excellent review works related to its treatment were published in the last two years; therefore, we would not multiply the information [7,8,9,10,11,12,13,14].

An important aspect of sewage sludge utilization is that it is possible to obtain a commercial end product meeting the qualitative requirements of the market, e.g., related to building materials. For example, the dewatered primary sludges have been proposed as fillers in aggregated concrete production in the energy-saving building construction industry [15,16]. Sewage sludge was also introduced into the manufacturing of mortar products in raw form, but it decreased the durability of materials, resulting from the high content of heavy metals [17]. When applied as a water replacement in mortar mixes it increased the water absorption and reduced the shrinkage of mortar mixes [18]. During the production of bricks, sewage sludge reduced the compressive strength and enhanced the water absorption of final products [19]. A reduction in mechanical performance was also noted when sewage sludge was introduced into the preparation of ceramic floor tiles [20]. In general, in almost all works, the authors mentioned that the sewage sludge could be introduced into the manufacturing of various building materials in reasonable amounts, but its impact on the properties is not very straightforward and could be comprehensively investigated.

Therefore, it is justifiable to search for the new possibilities of sewage sludge utilization in building materials. Sewage sludge can be considered as a raw material to fabricate lightweight aggregate (LWA). The production of LWA is one innovative method for sludge utilization, which can be widely used in building materials or water-treatment filter materials [21]. So far, research has been conducted on LWA made from sludge or sludge ash. Some previous studies have revealed that sludge sintered at 1000–1200 °C used as a raw material could result in porous LWA with a high compressive strength and proper density [22,23].

In the presented paper, we also investigated the manufacturing of lightweight aggregates as a potential method of sewage sludge utilization. This waste material was mixed with fly ash and waste clay generated by limestone mining through granulation in the intensive mixer. Then, a sintering process was applied to convert the obtained granules into LWAs. The process presented in this paper was described in the Polish patent application no. 431113 developed by the Central Mining Institute [24]. In preliminary works, we determined the optimal sintering temperature and time as 1150 °C and 10 min, respectively. In the presented work, the impact of sewage sludge:clay ratio, as well as the size of the intensive mixer, was investigated. The material’s physical and mechanical properties, i.e., bulk and particle density, water absorption, and crushing strength at various influential factors, such as materials proportion and mixer size, are investigated in this paper. The production of artificial LWAs from sewage sludge, fly ash from power plants and waste clay in this study provides a high potential for reusing significant SS and waste materials, consequently resolving the disposal problem of these waste streams.

## 2. Materials and Methods

### 2.1. Materials

Clay (C), which was the waste material generated during limestone mining, was obtained from the limestone mine “Czatkowice” (Krzeszowice, Poland). Fly ash (FA) was provided by Tauron Production (Katowice, Poland) and was obtained from fluidized bed boilers. Sewage sludge was received from the Gigablok sewage treatment plant, which belongs to Katowicka Infrastruktura Wodociągowo-Kanalizacyjna sp. z o. o., while the object was operated by Spółka Katowickie Wodociągi S.A. Characteristics of the used sewage sludge are presented in Table 1.

### 2.2. Granulation of Lightweight Aggregates

The LWA granulation was performed in three types of intensive mixer from IdeaPro (Poznań, Poland). Mixers were equipped with a star-belt type stirrer rotating in the opposite direction to the mixer pan rotation, enabling the disintegration and fragmentation of agglomerates. In this type of mixer, 90% of energy is used for the mixing of the material. They enable the efficient mixing of components with any consistency. A high degree of homogeneity can be achieved in a relatively short time. This is essential when low-volume additives are introduced into the material. Moreover, due to their construction, intensive mixers allow the simultaneous mixing and granulation of the material. As a result, granules with the desired size and density may be prepared by adjusting the process parameters. Compared to other methods, granulation in an intensive mixer eliminates the defects, such as the nut-shell structure of granulates. Generally, this process results in the efficient mixing of compounds, providing a homogenous composition for the whole batch of material, and enables efficient granulation. Compared to the disc or drum pelletizer, granulation in the intensive mixer runs in the whole volume of the processed material, and does not require complex control [25,26].

**Figure 1:**
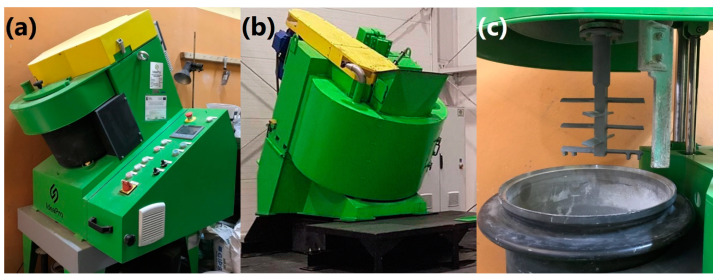
Intensive mixers with (**a**) 30 L and (**b**) 500 L agitator bowls, and (**c**) star-belt type stirrer present in applied mixers.

To investigate the effect of upscaling, LWAs were prepared in the laboratory and in industrial mixers with volumes of 5, 30, and 500 L (see Figure 1). Preliminary tests were performed for all mixers’ volumes, and then the final tests were conducted for the selected materials. During granulation, the stirrer and agitator bowl were counter-rotating, which resulted in high values of linear velocity around 20 and 30 m/s, respectively, for the 30 and 500 L agitator bowls.

Three composition variants were analyzed, which are presented in Table 2. Fly ash is commonly known as a component in the manufacturing of lightweight aggregates. Therefore, its content was fixed at 20 wt.%, and the primary goal of the performed experiments was to examine the varying contents of clay and sewage sludge, which, as waste materials, could be considered as exciting alternatives for conventionally applied raw materials.

### 2.3. Sintering of Lightweight Aggregates

Previously granulated samples were fired at a tube furnace with rotating reactor PRS 150 × 1500/110/OBR from Czylok (Jastrzębie-Zdrój, Poland). The basic technical parameters of the furnace are as follows:

- maximum operational temperature—1100 °C;
- three heating zones;
- length of the heating zone—1500 mm;
- internal diameter—150 mm.

The furnace is equipped with a three-zone heater and a horizontal reactor. Each heating zone is equipped with a separate heating controller and has a heating power of 6 kW (total power of 18.5 kW). The used furnace is presented in Figure S1 (Supplementary Materials).

The rotary tube furnace was selected for the experiment as the most suitable. It allows for conducting the process in a continuous manner (similar to industrial conditions) without stopping loading the raw material or receiving the product. Such a solution allows us to obtain stable process conditions that are unchanging in time. In such a reactor, it is possible to control the atmosphere continuously and, if necessary, change it in real-time. Additionally, the ease of implementing the post-reaction gas discharge affecting the firing process’ course has a significant impact on the choice of furnace type. The ease of the adjustment and change of the critical process parameters during the process was an additional advantage. In general, the process combined two operations: the firing of combustible fractions (sewage sludge) causing porosity, and the sintering (consolidation) of the granulate. Sludge firing is a highly exothermic process (it is possible to autogenously, locally, increase the furnace) at the beginning of the working zone when the temperature exceeds the ignition temperature. This process is accompanied by an intensive exhaust gas discharge, which must be removed from the working area as it will restrict the access of oxygen and thus inhibit the firing process. However, it should be remembered that removing the flue gases too quickly can result in excessively high oxygen supply and a temperature rise. Only in the rest of the reactor does the proper sintering of the granules take place. Therefore, it becomes crucial to be able to selectively control the parameters of individual heating zones in order to be able to carry out the process correctly. In the firing process of this type of granules, the total number of granules is subjected to the process simultaneously. The temperature and firing time of combustible fractions (I zone of the furnace), the temperature and sintering time of the specific granules (II and III zone of operation), the reactor’s rotational speed, the working inclination, the atmosphere, method, and quantity of air supplied to the process, the method of exhaust gas removal, and the method and speed of product cooling must all be selected so that the combustible components can be fired first and only then sinter the material. Failure to follow the sequence of processes can significantly increase the process’ time and cost and affect the product’s final properties. Therefore, to correctly carry out the process, it becomes necessary (often by an experimental method) to select all the relevant parameters.

The furnace’s reactor consists of a heat-resistant steel (type 1.4841—X15CrNiSi25-21) pipe with dimensions of 159 × 6.3 mm and a length of 3000 mm. Inside the reactor pipe, along its entire length, a forming band is made of an 8 mm heat-resistant rod with a 48 mm pitch (distance between coils, 40 mm). The reactor is mounted on supports and driven by a gear motor with a gear ratio of 62, via a chain (chain 12B) reduction gear with a gear ratio of 0.267. The reactor’s rotational speed at the nominal engine speed is 3.74 rpm, which, with a web stroke of 48 mm, gives the material residence time of about 120 min. The motor (gear motor) adjustment is in the range of 25–110% of the nominal engine speed—i.e., 0.935 to 4.11 rotations of the reactor per minute. The reactor is sealed with Teflon butt seals, and constant pressure is realized through a spring system mounted at the sintered raw material receiving unit. The angles of inclination of the furnace and the reactor, and the other working elements of the assembly, relative to the ground plane, are adjustable in the angle range from 0 to 4°. Tilting is carried out using a hydraulic system consisting of a hydraulic siphon and a position lock installed on the loading side (batch hopper).

During sintering, the raw materials are dosed to the working reactor with an automatic screw feeder (Figure S1b). The main working element of the feeder is a screw with a working diameter of 60 mm and a stroke of 40 mm, driven by the MRA 40 gear motor with a gear ratio of 64 through a chain transmission (chain 8B) with a gear ratio of 0.217 (z1 = 60, z2 = 13); the feeder is equipped with a reinforced connection to the reactor which is also the basis of the entire reactor system with process gas connections. The feeder tank is made in a funnel with a capacity of 18 dm^3^ made entirely of stainless materials with an installed charge mixer driven by a chain gear from a screw driver. The tank is equipped with a batch window for loading the batch, sealed with a silicone gasket with a quick clamping closure. There is a rectangular sight glass for observing the sidewall’s charge level on the operator’s side.

At the end of the working reactor, the product collection unit consists of a receiving chamber made in the form of a funnel and suspended into the reactor, in which the reactor connector pipe is mounted. All these elements are made of stainless steel. The chamber is pressed against the reactor with a system of 4 springs. A detachable flange is mounted on the wall opposite the inlet to access the reactor and the collection tank. There are two 25 mm visors in the flange, a gas discharge stub, and a measuring thermocouple socket for temperature measurement in the second and third heating zones of the furnace working reactor chamber. Figure S2 (Supplementary Materials) shows a view of sintered granules during kiln firing.

The sintering process was conducted at 950 °C in an air atmosphere. The inclination of the working reactor was about 2°. Initially, the rotational speed for the so-called “quick tests” was 4.28 rpm, which gave a residence time of the granules in the hot zone of about 7.3 min (the variant was used for tests). The high reactor speed and many granules simultaneously staying in the chamber’s hot zone caused an intensive process of burning out the flammable fractions. A sharp rise in temperature on the feeder side was observed, and there was a real threat of ignition of the granules in the feeder. This phenomenon also caused an uncontrolled increase in temperature inside the reactor, which threatened to eliminate control over the process. The granules obtained in the “quick test” had a non-homogeneous internal morphology, clearly showing underburned components (bright outer border, darker areas inside). In order to improve the parameters of the granules, both the speed of the granules being fed (the number of granules fed in a unit of time) to the reactor and the rotational speed of the reactor (to 2.15 rpm) were reduced, which, at a 48 mm forming ribbon stroke, doubled the time of staying in the hot zone of the furnace to about 14.5 min. This allowed us to avoid uncontrolled temperature rise through the less dynamic firing process, and the internal structure of the granules was more homogeneous (gray color).

### 2.4. Measurements

The preliminary tests aimed at evaluating the upscaling effect included determining the particle size of the prepared granulates and the bulk density and crushing resistance of sintered LWAs.

The particle size distribution of prepared granules was determined according to the PN-EN 933-1 standard [27] using a LPzE-4e siever from MULTISERW-Morek Jan Morek (Brzeźnica, Poland) with sieves characterized by the following openings: 2, 4, 6, 8, 10 and 12 mm.

To determine the bulk density of the prepared LWAs, they were placed in a 1 L polypropylene beaker and weighted using the electronic balance.

The obtained aggregates were subjected to compression tests, and their crush resistance was determined based on the PN-EN 13055-1 standard [28]. According to the standard, crushing resistance tests can be performed for aggregates with a grain size from 4 to 22 mm, characterized by a bulk density higher than 150 kg/m^3^. Therefore, the fraction below 4 mm was removed from the samples prepared for testing individual aggregates. On the other hand, individual large granules in the material under the test volume could cause a false reading of the force value needed for the piston’s appropriate depth in the material under test. The tested aggregates were subjected to mechanical tests consisting of the uniaxial compression of granules in a steel cylinder on a testing machine with a maximum force of 10 kN.

Values of aggregates’ crushing resistance (C) were calculated according to the following Formula (1):

C = (L + F)/A(1)

where C is crushing resistance, MPa, L is force exerted by the piston, N, F is the force needed to sink the piston, N, and A is the piston surface, mm^2^.

The final tests conducted for the selected materials based on the preliminary evaluation included determining the specific weight, open-cell content, LWAs’ microstructure, water uptake, and changes of these parameters after the aging of materials in different environments.

The specific weight of selected LWAs was determined using Ultrapyc 5000 Foam gas pycnometer from Anton Paar (Warszawa, Poland). The following measurement settings were applied: gas—helium; target pressure—18.0 psi; flow direction—sample first; temperature control—on; target temperature—20.0 °C; flow mode—monolith; cell size—medium, 45 cm^3^; preparation mode—flow, 0.5 min; the number of runs—5.

The microstructure of lightweight aggregates was evaluated using the Olympus SZ 11 microscope (Olympus Corporation, Tokyo, Japan). This enables geometrical measurements, and thanks to the backlighting of light guides, it enables a broader range of light adjustment. It was additionally equipped with a Nikon camera for image recording.

The water uptake of the prepared LWAs was studied for seven days. The samples were put into distilled water, then taken out and weighed after 24, 48, 72, 96, 120, 144, and 168 h. Water uptake was determined according to the following Formula (2):

W = (m_t_ − m_0_)/m_0_ × 100% (2)

where m_t_ is the mass of the sample after immersion, g, and m_0_ is the initial mass of the sample, g.

After the water absorption tests, when samples were conditioned in water for seven days, they were subjected to freeze–thaw tests. Ten cycles of freezing samples to −10 °C and thawing at a room temperature of 25 °C were performed for each sample. The time of each cycle equaled 4 h. After the test, the mechanical properties of LWAs were measured to determine their resistance to freezing.

To determine the environment’s impact on prepared lightweight aggregates, samples were conditioned for seven days in 10% solutions of NaCl, H_2_SO_4_, and NaOH. After the test, the mechanical properties of LWAs were measured to determine their resistance to different pH values. Moreover, the structure of LWAs was analyzed with optical microscopy.

## 3. Results and Discussion

### 3.1. The Particle Size Distribution of Granulates

**Figure 2:**
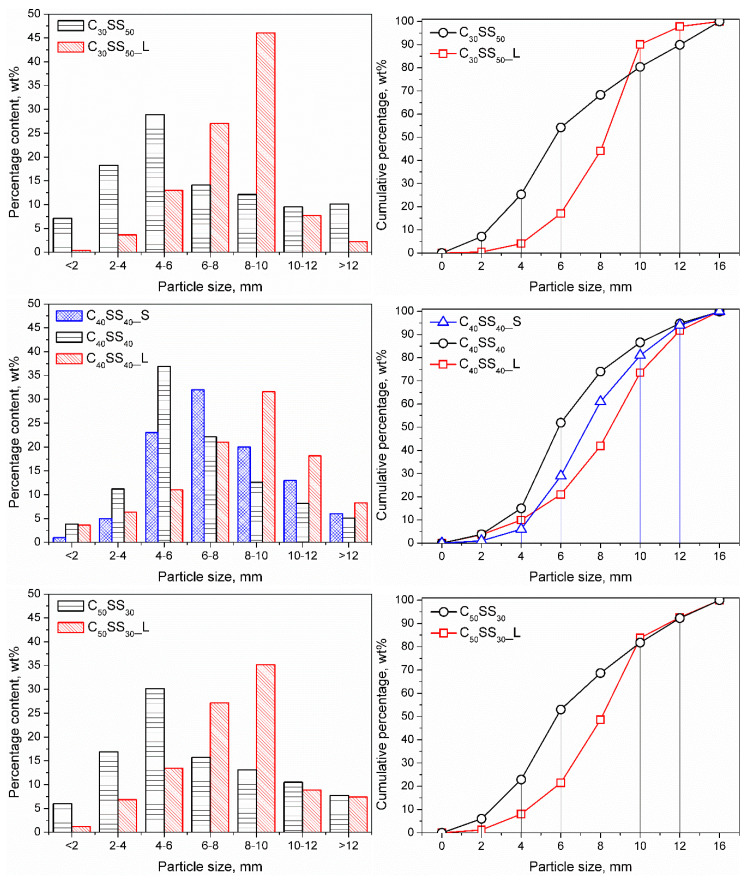
The influence of composition and agitator bowl volume over the particle size distribution of the prepared granulates.

Figure 2 presents the particle size distribution of prepared granulates depending on the applied composition and size of the agitator bowl. For samples prepared in the 30 L mixer, the particle size distribution is rather broad. Each fraction between 2 and 10 mm accounted for at least 11% of the total mass, while the only share of the 4–6 mm fraction exceeded 22 wt.%. Therefore, the homogeneity of these materials was rather low.

The upscaling of the process and the increasing of the mixer volume to 500 L resulted in the rise of the average particle size with a simultaneous increase in granulate homogeneity. The particle size distribution was more narrow, with dominating fractions of 6–8 and 8–10 mm, which accounted for 73.05, 52.56, and 62.30 wt.%, respectively, for clay contents of 30, 40, and 50 wt.%. In the case of the equal content of clay and sewage sludge, the 10–12 mm fraction was quite significant since it accounted for 18.16 wt.% of the total mass. The content of fractions lower than 4 mm did not exceed 10 wt.%, which can be considered beneficial from the application point of view.

On the other hand, the reduction of agitator size also resulted in a narrowing of particle size distribution. However, the most significant was a fraction of 6–8 mm (31.90 wt.%), with high shares of 4–6 mm (23.17 wt.%) and 8–10 mm (19.93 wt.%) fractions. These fractions accounted for 75 wt.% of the total mass.

It can be seen that particle size is influenced by composition and processing parameters (mixer size, hence values of linear velocity inside the bowl). Therefore, it is possible to obtain granulation with the desired average particle size and distribution by appropriately adjusting these factors. Such an effect should be considered beneficial from the application point of view because it allows the separation of granulates and reuses the unnecessary fraction to obtain a material with the desired properties. It gives the possibility of granulation production with a particular size, as requested by the customer, without the necessity of storing fractions hardly or not sold.

### 3.2. Preliminary Tests of Sintered Aggregates

**Figure 3:**
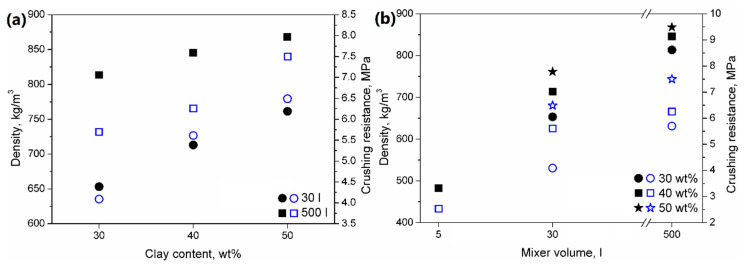
The effect of (**a**) clay content and (**b**) mixer volume on the (●,■,★) bulk density and (○,□,☆) crushing resistance of obtained aggregates.

In Figure 3, there are presented the values of the bulk density and crushing resistance of the prepared LWAs. Increasing the clay content significantly affected the aggregates’ density, which was associated with a more concise and less porous structure. Such an effect was associated with moisture content differences between clay (~20 wt.%) and sewage sludge (~80 wt.%). Its higher value resulted in the enhanced generation of volatiles during the sintering of LWAs, simultaneously increasing their porosity [29]. Moreover, the density of LWAs was strongly affected by the mixer volume due to differences in the linear velocities and values of stress acting on the material during granulation. For higher volumes, more compact structures were obtained, which resulted in higher densities.

It can be seen that crushing resistance is strictly associated with the material’s density and clay and sewage sludge content. Generally, the mechanical performance of all porous materials (e.g., aggregates), insulation materials, polymeric foams and others, is strongly dependent on their apparent density, which determines the solid and gaseous part in the material [30]. The increase in clay content caused the enhancement of the mechanical performance of LWAs, which was noted for both types of applied mixers, independently of their volume. Such an effect was related to the lower moisture content of clay compared to sewage sludge, which resulted in the lower amount of volatiles evaporated during sintering and the lower porosity of the LWAs. The higher share of solid material in aggregates increased the force which could be withstood by the material. Therefore, during the application of sewage sludge as a raw material for lightweight aggregates production, a compromise between the mechanical performance and the amount of introduced waste material (and thus cost) should be found.

Nevertheless, due to the higher values of the LWAs’ density, the manufacturing process’ upscaling led to mechanical performance enhancement. The increase in the mixer volume from 30 to 500 L enabled the increase in sewage sludge content in the LWA’s composition without the deterioration of the crushing resistance. Samples C_30_SS_50__L and C_40_SS_40_ showed very similar values of crushing resistance, similar to C_40_SS_40__L and C_50_SS_30_ samples. A similar effect, associated with the higher shear forces (here caused by the increase in the mixer size) was noted by Rahmanian et al. [31,32].

The obtained crushing resistance values are auspicious because they are significantly higher than the values for many commercially available LWAs present on the market. Exemplary values of this parameter for various LWAs are:

- Arlita—0.98 MPa;
- Lytag—0.43 MPa;
- LECA and Ardelite—0.09 MPa;
- Geokeramzyt Matrix—0.8 MPa;
- LECA Gniew—0.7–4.0 MPa.

Generally, it can be seen that the applied waste materials may be efficiently applied in the manufacturing of lightweight aggregates with comparable or higher mechanical properties than commercial LWAs.

Because of the highest crushing resistance values, the LWAs prepared in a 500 L mixer were selected for the final tests. Moreover, the application of a 500 L mixer enables the manufacturing of LWAs on a near-industrial scale. Therefore, the presented results of the research works could be transferred to the industrial manufacturing of LWAs.

### 3.3. Final Tests of Sintered Aggregates Prepared in a 500 L Intensive Mixer

A previous section of the manuscript presented the bulk density and crushing resistance of lightweight aggregates prepared in a 500 L intensive mixer. Figure S3 (Supplementary Materials) presents photographs of sintered aggregates before and after the crushing resistance tests for a more comprehensive analysis of their performance. They confirm the results of the particle size distribution of the granules before the sintering process. The lowest content of the fraction below 4 mm was noted for C_30_SS_50_ samples, while the granulate C_40_SS_40_ showed the highest fraction content, which can also be observed in the presented photographs.

**Figure 4:**
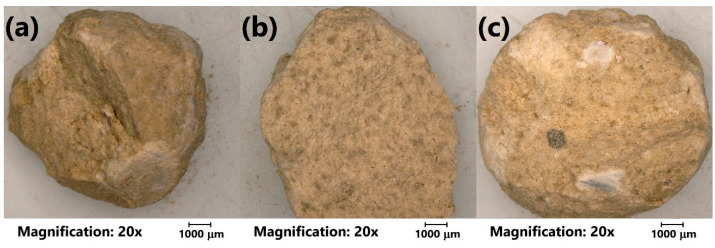
Images of (**a**) C_30_SS_50_, (**b**) C_40_SS_40_, and (**c**) C_50_SS_30_ aggregates obtained with optical microscope.

**Figure 5:**
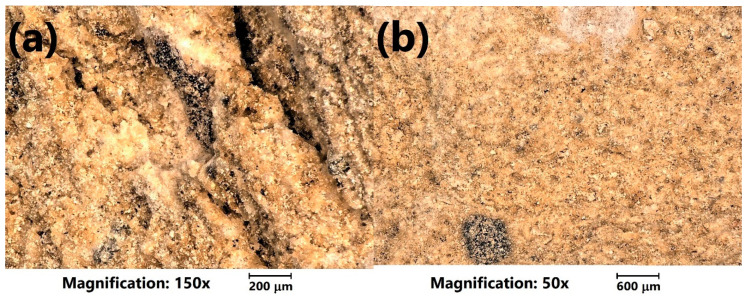
Images of the (**a**) C_30_SS_50_ and (**b**) C_50_SS_30_ LWAs’ surfaces obtained with an optical microscope at higher magnifications.

Moreover, for a more detailed investigation, the analysis of the LWAs’ structure with optical microscopy was performed. Figure 4 shows the initial appearance of the prepared LWAs. It can be seen that the porosity of the prepared materials was more significant for the higher content of sewage sludge, which can be seen in the magnified images in Figure 5. The sample of C_30_SS_50_ shows noticeable linear cracks, which result from the higher amounts of volatiles generated during sintering, compared to the C_50_SS_30_ material.

**Figure 6:**
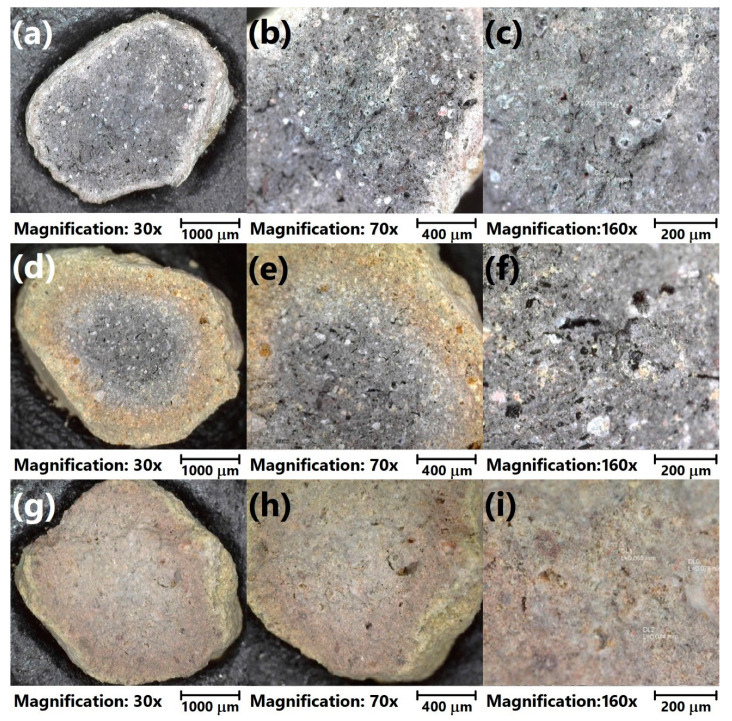
Images of fracture surfaces of (**a**–**c**) C_30_SS_50_, (**d**–**f**) C_40_SS_40_, and (**g**–**i**) C_50_SS_30_ LWAs under different magnifications.

Figure 6 presents the morphology of analyzed LWAs after the crushing resistance tests. The presented photographs confirm the obtained values of density and assumptions based on the photographs showing undestroyed material. It can be seen that the porosity of LWAs is the highest for the 30 wt. % content of clay, while for higher contents, a more concise structure was noted. Such an effect is associated with the evaporation of volatiles during the sintering of prepared granulates.

Moreover, noticeable differences in appearance are related to the color of fracture surfaces. Generally, fracture areas consist of a darker grey core and a lighter shell. The size of the core is significantly bigger for higher contents of sewage sludge. For sample C_50_SS_30_, a darker core is hardly present. The inhomogeneous appearance of aggregates might indicate the incomplete sintering of the material. The elongation of this process could increase the material’s homogeneity and porosity. As a result, the chemical resistance of the aggregates could be enhanced, but at the same time, it could unfavorably affect its mechanical performance [33].

Moreover, sintering is quite an energy-consuming process due to the high temperatures, so its elongation would noticeably affect its economic aspects. The rise of the process temperature could cause a similar effect. However, except for the increase in costs, it could generate a fire threat. Therefore, depending on the desired properties of the final LWAs, the compromise between cost (the content of low-cost waste–sewage sludge, temperature, and time of sintering) and performance should be found. The influence of the sintering parameters, e.g., time and temperature, on the properties of prepared materials, would definitely be the topic of our further studies. On the other hand, aggregates presented in the literature [31] and commercially available examples often show an inhomogeneous structure, which can be seen in the Liapor material shown in Figure S4 (Supplementary Materials). Moreover, compared to the commercial Liapor aggregates, the prepared materials are characterized by their significantly lower content of macropores.

**Figure 7:**
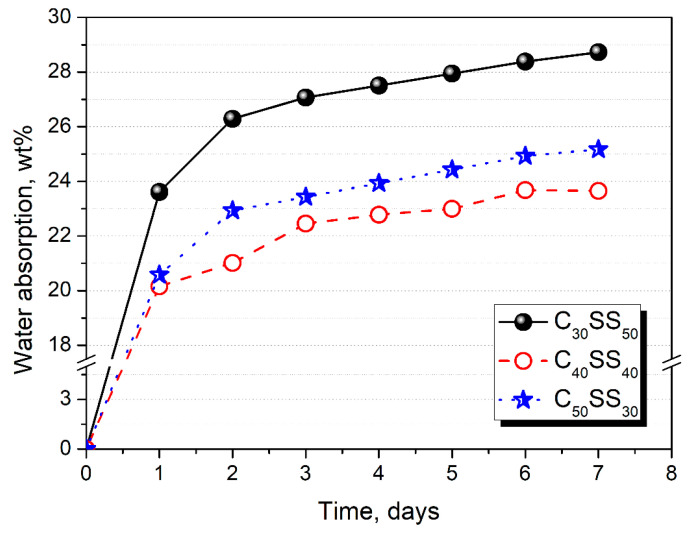
Water absorption of LWAs prepared in a 500 L mixer.

In Figure 7, there are presented plots showing the water absorption of LWAs depending on their composition. It can be seen that significantly higher values were noted for materials with lower contents of clay and higher contents of sewage sludge. Such an effect was related to the differences in porosity between particular samples, especially regarding open pores, which enable the penetration of water inside the granulates. The impact of composition on the porosity can also be seen in Figure 4, and was confirmed by differences in the density of materials.

After water absorption tests, the LWAs were subjected to freeze–thaw tests, which were aimed at assessing the resistance of the prepared materials to freezing. The prepared LWAs were also immersed in 10% solutions of NaCl, H_2_SO_4_, and NaOH for seven days. These experiments aimed to investigate the influence of atmospheric conditions and different environments on the structure and mechanical properties of prepared materials, which is essential from the application point of view. Such tests are commonly applied during the investigation of aggregates and concretes [34].

**Figure 8:**
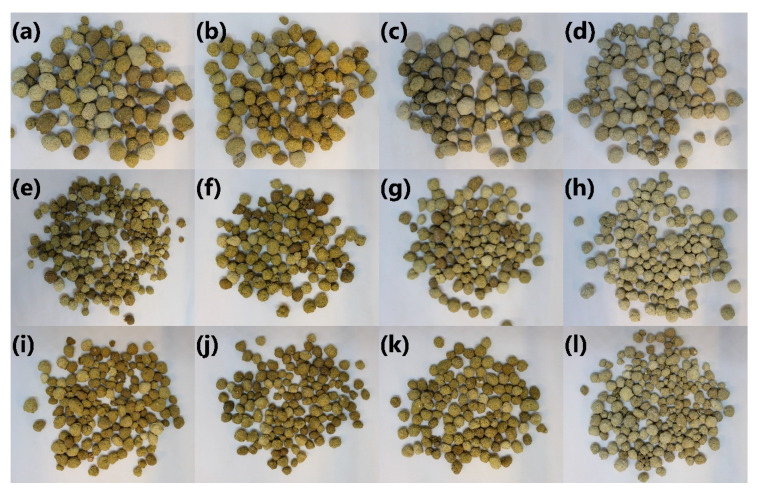
Appearance of (**a**–**d**) C_30_SS_50_, (**e**–**h**) C_40_SS_40_, and (**i**–**l**) C_50_SS_30_ LWAs (**a**,**e**,**i**) initially, and after immersion in (**b**,**f**,**j**) NaCl, (**c**,**g**,**k**) NaOH, and (**d**,**h**,**l**) H_2_SO_4_ solutions.

Figure 8 presents the appearance of LWAs initially and after treatment with different solutions. Sample C_30_SS_50_ was paler than materials prepared with a higher share of clay. Such an effect may be associated with the material’s higher porosity, which increases the surface’s roughness, enhancing the light reflection. As a result, the lightness of the material was higher. Such an effect is often noted in powders [35]. Due to the highest lightness, the sample C_30_SS_50_ was most sensitive towards color change after immersion in NaCl solution, which resulted in a more yellow and green color. On the other hand, immersion in a sulfuric acid solution resulted in the fading of LWAs’ color. The most substantial effect was noted for higher clay contents, which suggests chemical reactions during immersion.

**Figure 9:**
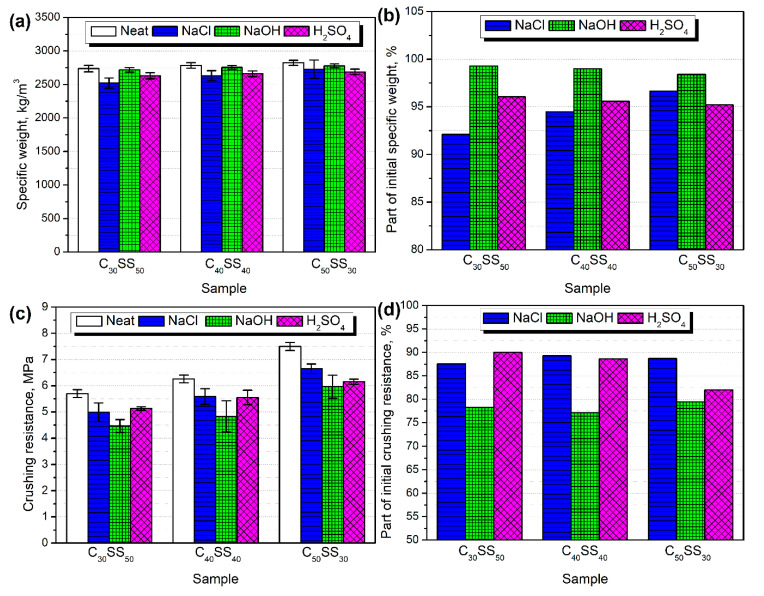
The impact of different treatments on the (**a**,**b**) specific weight and (**c**,**d**) crushing resistance of the analyzed LWAs.

In Figure 9, there are presented the values of the LWAs’ specific weights. It can be seen that they were sensitive to different environments. The most significant decrease, 8%, was noted when samples were immersed in the sodium chloride solution. This effect was especially pronounced for higher contents of the sewage sludge in LWAs. On the other hand, higher loadings of clay increased the sensitivity of LWAs to alkaline and acid environments.

**Figure 10:**
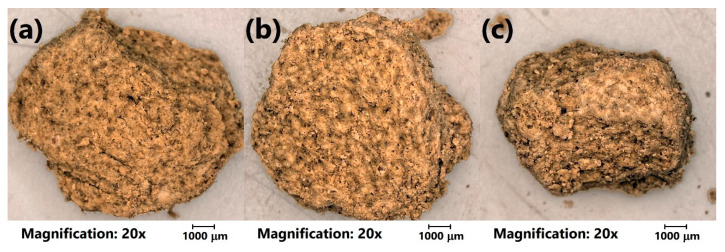
The appearance of the C_40_SS_40_ sample’s surface after immersion in (**a**) NaCl, (**b**) NaOH, and (**c**) H_2_SO_4_ solutions.

Figure 9 presents the values of the crushing resistance of the prepared lightweight aggregates after conditioning in various media. Independently of the composition, deterioration of the mechanical performance was observed. Such an effect was probably associated with the changes in the chemical structure of LWAs, which is also suggested by changes in the LWAs’ appearance and specific weight. A slightly lower decrease was noted for milder conditions—sodium chloride solution. More aggressive environments resulted in the higher deterioration of crushing resistance, which can be seen mostly for the NaOH treatment. In the case of the C_50_SS_30_ sample, the immersion in H_2_SO_4_ also caused a very significant decrease in crushing resistance. Such an effect is associated with the chemical reactions and enhanced porosity of the structure, which can be seen in Figure 10, presenting the appearance of the C_40_SS_40_ sample after aging in different environments.

It can be seen that for all types of applied solutions, the surface of the aggregates is very porous, with significant amounts of voids. As mentioned above, this can be associated with chemical reactions during immersion and the leaching of different components of analyzed materials. According to Smeck and Novak [36], the incubation of clays in sulfuric acid resulted in the release of noticeable amounts of magnesium, potassium, and aluminum cations from different types of clays. Silicone and iron were released in slightly smaller amounts. Similar observations related to acid treatment were made by Carroll and Starkey [37], who used hydrochloric acid. They detected significant amounts of aluminum and iron oxides released for different types of clays, followed by magnesium and silicon oxides. At the same time, they analyzed clay treatment with NaOH and NaCl solutions. In these cases, they mostly detected silicon, aluminum, and calcium oxides. However, these were in different amounts. The NaCl treatment resulted in a release of cations that was as much as 50 times smaller (in parts per million) than the HCl and mostly NaOH treatments, which was the most aggressive for most of the clays. These results may confirm the significant drop in the mechanical performance of LWAs investigated in the presented work.

The noticeable difference between the acid and alkali treatments may also be related to the changes in the chemical character of LWAs. As presented in Figure 8, after immersion in H_2_SO_4_, the LWAs changed color noticeably, while for the NaOH treatment, this effect was not as pronounced. According to the works cited above [36,37], acid treatment results in the release of exchangeable ions such as magnesium or potassium, indicating changes in the chemical structure of LWAs, while alkali treatment causes mainly the partial removal of material without any replacement. It also explains the deterioration of the mechanical performance of LWAs.

## 4. Conclusions

The presented research work aimed to evaluate the possibility of applying sewage sludge in the manufacturing of lightweight aggregates. Sewage sludge is currently relatively rarely used in industrial processes. Therefore, it has a very high availability and low price. The obtained results indicate that sewage sludge application may lead to a relatively broad spectrum of apparent density and crushing resistance in LWAs. Moreover, the resistance of prepared LWAs to different environments has been determined. The presented results indicate that sewage sludge can be introduced into the manufacturing of LWAs as the main component, and as an auxiliary component. Its incorporation can be aimed at manufacturing material with the desired properties, e.g., with increased porosity. Considering its waste character and low price, the introduction of sewage sludge may be applied to find the compromise between a material’s performance and cost.

Further works related to this area should address the following issues:

- The level of sewage contamination. For higher contents of impurities, it may lead to the accumulation of harmful substances in the aggregates, which may result, e.g., in the migration of contaminants to the environment;
- The emissions of volatile organic compounds during the manufacturing of LWAs from sewage sludge. Sewage sludge is an odorous material, especially without the special treatment. Therefore, future research works should include the assessment of volatile organic compounds emissions, e.g., with the use of passive dosimetry. Moreover, the gases generated during sintering could also be monitored;
- The comprehensive analysis of the environmental impacts of the presented process, especially considering the water footprints, since sewage sludge contains high amounts of moisture. Moreover, the life cycle assessment of the process could include the eco-effectivity analysis;
- The influence of sintering parameters (temperature, time, atmosphere) on the performance of the resulting LWAs depending on their composition;
- Modification of the LWAs’ composition to develop the autothermic process, where the energy required for sintering would be obtained from the combustion of particular components.

## Figures and Tables

**Table 1:** Characteristics of sewage sludge.

Component	Content, wt. %
Moisture	80.64
Dry mass	19.36
**Component**	**Content, wt. % of dry mass**
Organic compounds	64.61
Volatiles	54.96
Ash	35.39
C	34.52
H	4.98
N	8.80
O	15.16
S, total	1.20
S, ash	0.05
S, combustible	1.15
P	3.68
K	0.50
Mg	0.92
Ca	2.34
Fe	5.23

**Table 2:** The composition of prepared granulates further sintered into lightweight aggregates (LWAs).

Component	Sample Code						
	C_30_SS_50_	C_30_SS_50__L	C_40_SS_40__S	C_40_SS_40_	C_40_SS_40__L	C_50_SS_30_	C_50_SS_30__L
Clay	30	30	40	40	40	50	50
Sewage sludge	50	50	40	40	40	30	30
Fly ash	20	20	20	20	20	20	20
Volume of mixer, l	30	500	5	30	500	30	500

## Supplementary Materials

The following are available online at https://www.mdpi.com/1996-1944/13/24/5635/s1, Figure S1: The photograph of (a) rotary tube furnace used in the presented study and (b) automatic screw feeder, Figure S2: The viewfinder in the furnace flange showing the sintered granules during kiln firing, Figure S3: Appearance of (a,b) C_30_SS_50_, (c,d) C_40_SS_40_, and (e,f) C_50_SS_30_ aggregates, (a,c,e) before, and (b,d,f) after the crushing resistance tests, Figure S4: The fracture surface of the commercially available Liapor aggregate.
