# Supplementary material for: Manufacturing of Lightweight Aggregates as an Auspicious Method of Sewage Sludge Utilization
Source: Materials (Basel). 2020 Dec 10;13(24):5635. doi: 10.3390/ma13245635 (PMC7764059; doi:10.3390/ma13245635)
Supplement: Supplementary file 1 [file materials-13-05635-s001.pdf]

materials

Supplementary Materials

Manufacturing of Lightweight Aggregates as an Auspicious Method of Sewage Sludge Utilization

Jerzy Korol [1,*], Marcin Głodniok [2], Aleksander Hejna [3], Tomasz Pawlik [4], Błażej Chmielnicki [5] and Jan Bondaruk [2]

[1] Department of Material Engineering, Central Mining Institute, Pl. Gwarków 1, 40-166 Katowice, Poland
[2] Department of Water Protection; Central Mining Institute, Pl. Gwarków 1, 40-166 Katowice, Poland; mglodniok@gig.eu (M.G.), jbondaruk@gig.eu (J.B.)
[3] Department of Polymer Technology, Gdańsk University of Technology, Narutowicza 11/12, 80-233 Gdańsk, Poland; aleksander.hejna@pg.edu.pl
[4] Faculty of Material Engineering and Metallurgy, Silesian University of Technology, Krasińskiego 8, 40-019 Katowice, Poland; Tomasz.Pawlik@polsl.pl
[5] Paint & Plastics Department in Gliwice, Institute for Engineering of Polymer Materials and Dyes, 50 A Chorzowska Street, 44-100 Gliwice, Poland; b.chmielnicki@impib.pl
* Correspondence: jkorol@gig.eu; Tel.: +48-32-259-2644Supplementary material

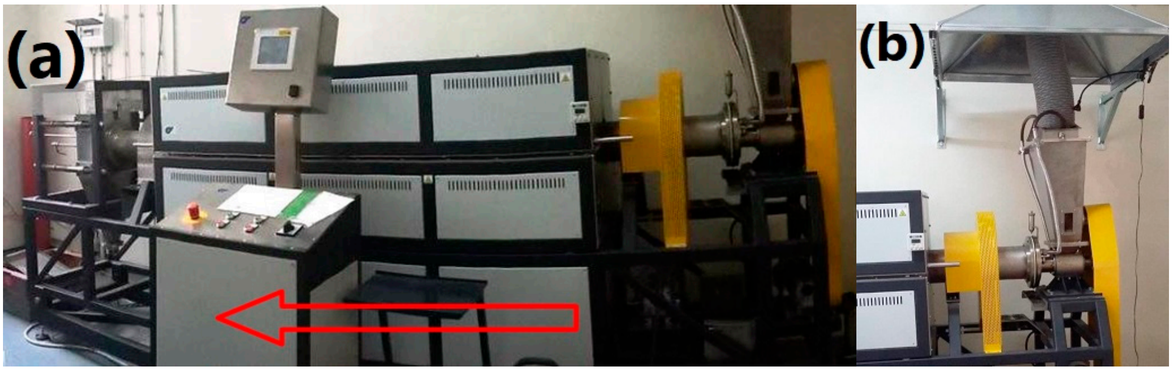

Figure S1. The photograph of (a) rotary tube furnace used in the presented study and (b) automatic screw feeder.

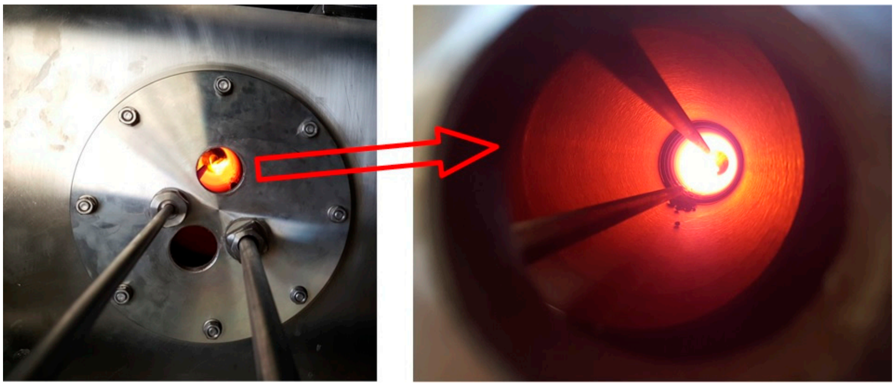

Figure S2. The viewfinder in the furnace flange showing the sintered granules during kiln firing.

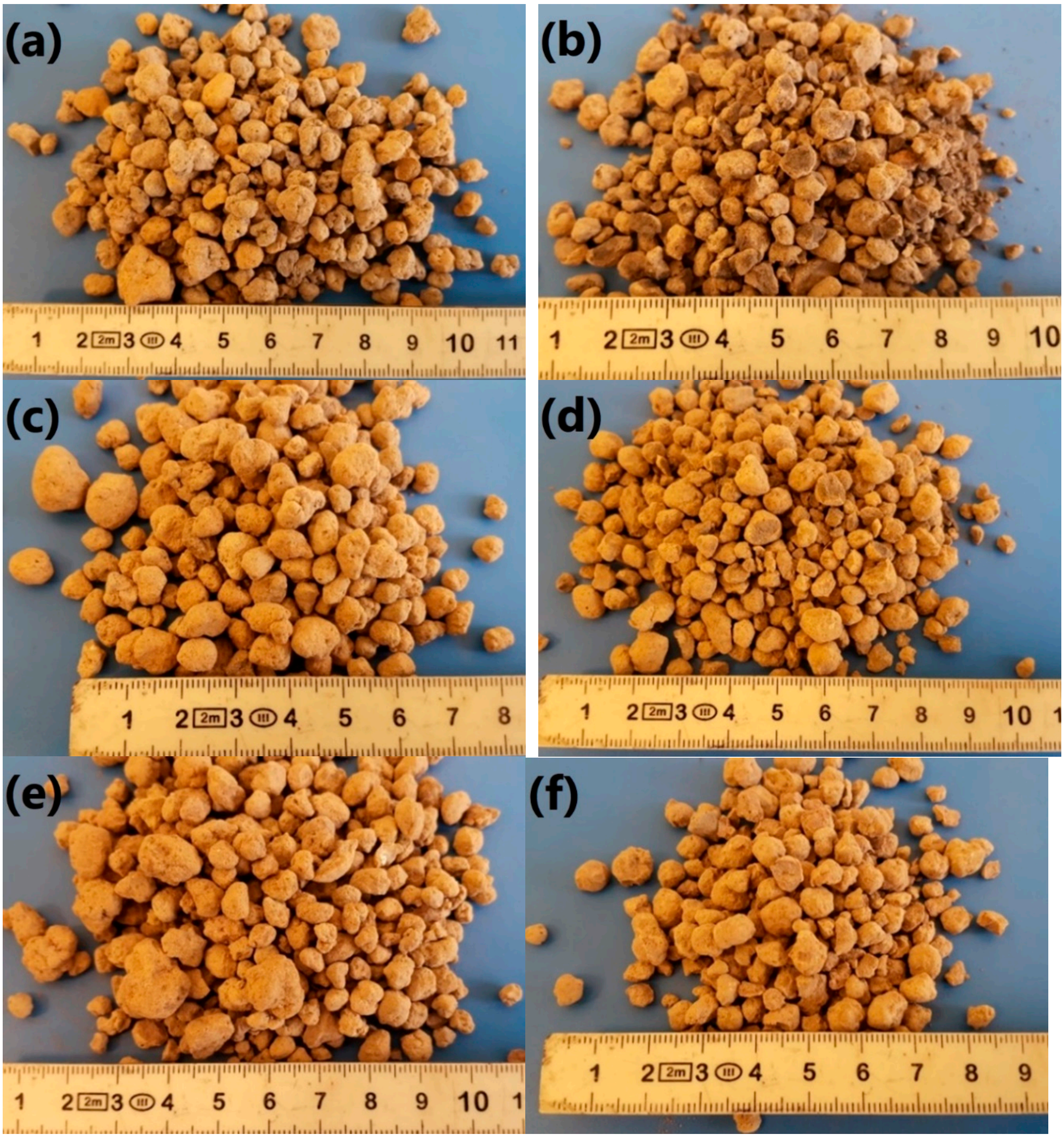

Figure S3. Appearance of (**a**,**b**) $C_{30}SS_{50}$, (**c**,**d**) $C_{40}SS_{40}$, and (**e**,**f**) $C_{50}SS_{30}$ aggregates, (**a**,**c**,**e**) before, and (**b**,**d**,**f**) after the crushing resistance tests.

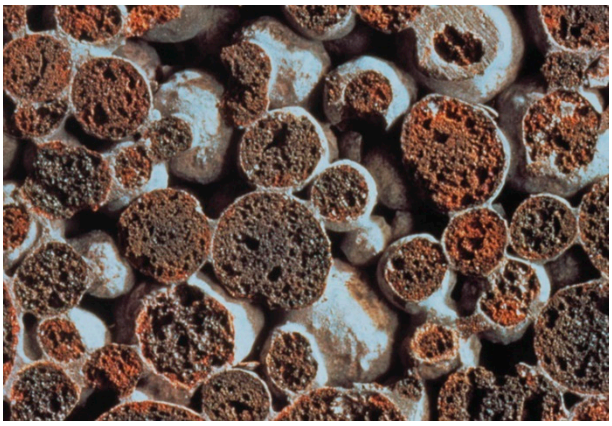

Figure S4. The fracture surface of the commercially available Liapor aggregate.
